# Isolation and Characterization of L-Asparaginase-Producing Bacteria from the Arabian–Persian Gulf Region: First Report on *Bacillus xiamenensis* ASP-J1-4 as a Producer and Its Potential Application

**DOI:** 10.3390/md23050194

**Published:** 2025-04-29

**Authors:** Ghofran M. Al-Harbi, Essam Kotb, Abeer A. Almiman, Mahmoud M. Berekaa, Salwa Alhamad, Nada F. Alahmady, Meneerah A. Aljafary, Nadiyah M. Alqazlan, Reem I. Alyami, Joud M. Alqarni, Ebtesam Abdullah Al-Suhaimi

**Affiliations:** 1 Department of Biology, College of Science, Imam Abdulrahman Bin Faisal University (IAU), P.O. Box 1982, Dammam 31441, Saudi Arabia; 2230500262@iau.edu.sa (G.M.A.-H.); nalahmadi@iau.edu.sa (N.F.A.); maljafary@iau.edu.sa (M.A.A.); nmalqazlan@iau.edu.sa (N.M.A.); 2200006031@iau.edu.sa (J.M.A.); 2 Basic and Applied Scientific Research Center (BASRC), Imam Abdulrahman Bin Faisal University (IAU), P.O. Box 1982, Dammam 31441, Saudi Arabia; 3 Department of Environmental Health, College of Public Health, Imam Abdulrahman Bin Faisal University (IAU), Dammam 31441, Saudi Arabia; mberekaa@iau.edu.sa; 4 Vice Presidency of Scientific Research and Innovation, Imam Abdulrahman Bin Faisal University (IAU), Dammam 31441, Saudi Arabia

**Keywords:** L-asparaginase, marine bacteria, marine drugs, antitumor, *Bacillus xiamenensis*, Arabian–Persian Gulf

## Abstract

L-asparaginase (L-ASNase) functions as a chemotherapeutic enzyme with antitumor properties. It facilitates the degradation of L-asparagine (L-ASN), a vital amino acid required for the proliferation of tumor cells. In this study, we have isolated 177 L-ASNase-producing strains from the aquatic environment of the Arabian–Persian Gulf. The most potent isolate, ASP-J1-4, was an endophyte recovered from the seablite *Suaeda maritima* and was molecularly identified as *B. xiamenensis* (accession number PQ593941). The enzyme purified through DEAE-Sepharose displayed a molecular weight of 37 kDa based on the SDS-PAGE profile and lacked detectable L-glutaminase (L-GTNase) activity. Optimal enzyme activity was at 40 °C and pH 9.0, with stability at pH 7–9. The maximum stimulation effect was found in the presence of Fe^3+^, Mn^2+^, and Na^+^ ions, respectively. The enzyme demonstrated a *V*_max_ of 35.71 U/mL and a *K*_m_ of 0.15 mM. Interestingly, ASP-J1-4 L-ASNase showed a dose-dependent inhibition against human colon carcinoma (HCT-116) and cervical Henrietta Lacks (HeLa) cell lines, with IC_50_ values of 15.42 µg/mL and 12.13 µg/mL, respectively. These findings collectively suggest a biocompatible, efficient, and robust enzyme for potential applications in tumor therapy after validation of in vivo studies and clinical trials. This study introduces the first deep screening program for L-ASNase-producing bacteria harboring in the Arabian–Persian Gulf region. In addition, it launches *B. xiamenensis* and other species as new sources of L-ASNase.

## 1. Introduction

The therapeutic potential of bacterial enzymes has been confirmed in several studies [1,2,3,4]. L-ASNase or L-ASN amidohydrolase (EC 3.5.1.1) is widely applied as an antitumor enzyme in treating lymphoblastic leukemia (ALL) and various malignant cancers. This enzyme facilitates the hydrolysis of L-ASN, a critical amino acid required by lymphoblasts, into ammonia and aspartic acid through deamination. Therefore, the therapeutic use of this enzyme hinges on its ability to break down L-ASN in the cerebrospinal fluid and serum. To support tumor proliferation, lymphatic tumor cells depend on a significant L-ASN supply sourced from diet and neighboring cells, continuously minimizing the concentration of L-ASN through administration of L-ASNase, resulting in lymphoblast cell death [5]. 

Colorectal cancer ranks among the most prevalent and lethal cancers globally across both sexes. It is the second main reason for cancer mortality in Saudi Arabia. Cancer cells cannot produce L-ASN synthetase. However, numerous studies have shown that L-ASNase effectively inhibits tumor growth in mice, rats, and humans by disrupting tumor-specific cells. Most colorectal cancer-related mortalities result from the formation of secondary tumors due to metastasis. Variations in L-ASN availability significantly impact the regulation of metastatic progression [6].

The integration of L-ASNase into food technology emerged following Swedish research highlighting the widespread presence of acrylamide in foods, usually consumed starchy foods subjected to frying, baking, or roasting. L-ASNase is expected to decrease free L-ASN levels in starches used in food production, thereby mitigating the risk of acrylamide formation, which is known to be both a tumorigenic and neurotoxic factor [7]. 

Bacterial L-ASNase has significant medical relevance and is utilized in treating ALL. For over four decades, the enzyme from *Escherichia coli* and *Erwinia chrysanthemi* has been routinely employed in leukemia therapies and continues to be utilized in treating ALL. However, it possesses L-GTNase activity, which can lead to severe adverse effects, including kidney issues, liver disease, pancreatitis, leukopenia, neurological seizures, and coagulopathy, and, recently, resistance to currently available commercial L-ASNases has emerged. Consequently, the discovery of new microbial sources that can produce L-ASNase free of L-GTNase while maintaining high therapeutic efficacy is of great importance [8].

There is an increasing interest in marine-derived bioactive compounds, including therapeutic enzymes, as novel agents for targeted cancer therapy [9], given the extensive biodiversity of the ocean, covering over 75% of the Earth’s surface. The above-mentioned study emphasizes the untapped potential of marine organisms such as algae, mollusks, and actinomycetes in yielding chemically diverse metabolites with significant anticancer properties. The work underscores how these bioactives contribute to emerging strategies in precision oncology, providing a strong foundation for the exploration of marine L-ASNase-producing bacteria like the one reported in our study. Furthermore, the biotechnological production and optimization of L-ASNase from various sources, including bacteria and fungi, has been extensively reviewed [10]. Their analysis underscores the enzyme’s pivotal role in industrial and therapeutic contexts, particularly in anticancer applications, where the depletion of L-asparagine induces apoptosis in tumor cells. The study also discusses some approaches aimed at enhancing enzyme stability and efficacy, aligning with our goal of identifying naturally potent, L-glutaminase-free L-ASNase candidates for safer clinical applications.

Recently, marine microorganisms have gained attention as L-ASNase producers, as they have unique characteristics and reduced secondary reactions [11]. Various species of *Streptomyces*, including *S. karnatakensis*, *S. venezualae*, and *S. longisporusflavus*, were investigated for their ability to produce this enzyme [8]. Despite the potential of marine bacteria, research in this area is still limited. Nonetheless, recently there has been a growing focus on such organisms due to their reputation as sources of antibiotics, antitumor agents, and other bioactive compounds. The Red Sea and the Arabian–Persian Gulf represent an exceptional marine ecosystem with a rich diversity of microbial communities with novel characteristics beneficial to the industry of biopharmaceutical companies, warranting further exploration. Numerous studies have highlighted the expected biological and medical implementations of marine *B. velezensis*, due to its ability to detoxify zearalenone in animal feeds, produce antimicrobial agents, biosurfactants, and exopolysaccharides that can stimulate apoptosis in malignant cells [6]. As far as we are aware, there is no existing research on L-ASNase production from *B. xiamenensis*. In addition, there is no previous deep screening for L-ASNase-producing bacteria from the Arabian–Persian Gulf region. The objective of this research was extended to purify, characterize, and in vitro apply the produced enzyme in the treatment of HCT-116 and HeLa carcinoma.

## 2. Results

### 2.1. Isolation and Screening of Marine Bacteria for L-ASNase Production

Exactly 178 L-ASNase-producing isolates were recovered from many marine organisms and sediments collected from the aquatic environment of the Arabian–Persian Gulf of Saudi Arabia by rapid plate assay. The most potent bacterial candidates were characterized by *16SrDNA* gene analysis and identified as *B. xiamenensis* ASP-J1-4 (from *Suaeda maritima*), *B. paralicheniformis* HOR-5 (from seahorse *Hippocampus kuda*) and SPF-4 (from Sponge #2), *Paenibacillus lautus* J6-1 (from shoreline soil), *Staphylococcus epidermidis* HOR-4 (from seahorse *Hippocampus*), *Proteus mirabilis* A-2 (from marine alga *Eucheuma*), *Macrococcus caseolyticum* K-3 (from *Planaria*), *Psychrobacter sanguinis* K2-2 (from sea squids *Sepia pharaonis*), and *Psychrobacter phenylpyruvicus* STF-1 (from seastar *Patiria miniata Asteroidea* #1). The *16SrDNA* gene fingerprints have been registered in the GenBank database (NCBI, NIH, USA) and given universal accession numbers (Table 1). Interestingly, most of them showed clinically desirable attributes, such as the extracellular productivity of L-ASNases and the lack of L-GTNase activity, except for isolates A-2 and SPBR-6 (Table 1).

**Figure 1:**
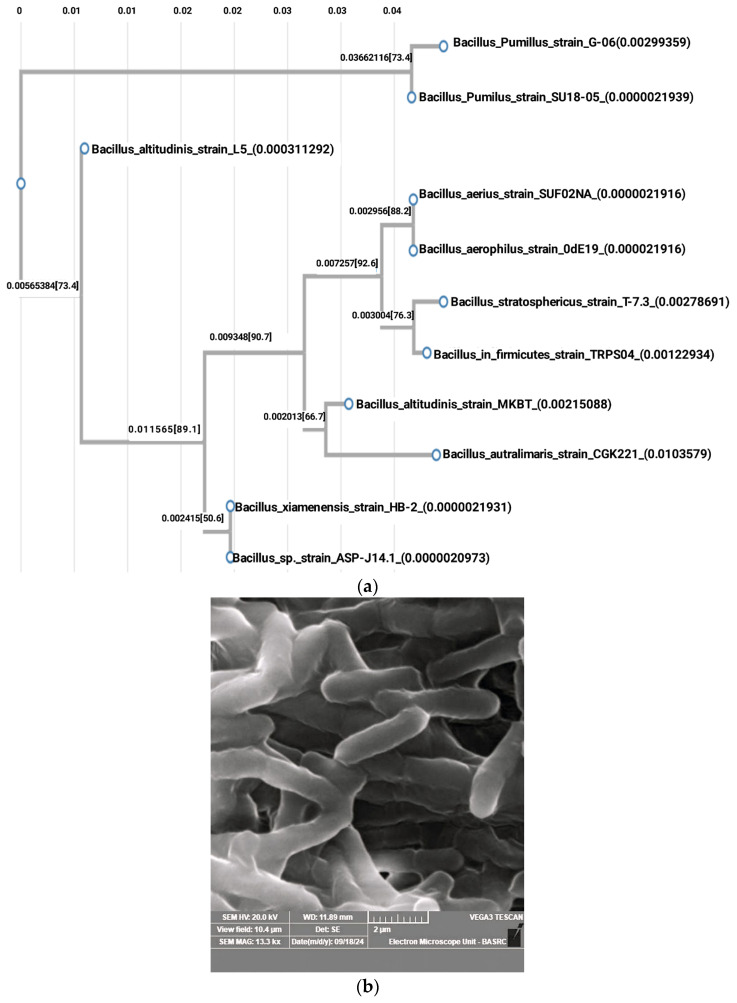
Phylogenetic tree of selected isolate *B. xiamenensis* ASP-J1-4 (accession number PQ593941) (**a**) with SEM micrograph showing the bacterial cells (**b**).

The most potent isolate, ASP-J1-4 (53.20 U/mL), was an endophyte recovered from *Suaeda maritima* collected from Jubail City. Its *16SrDNA* gene sequence showed 99.86% similarity with *B. xiamenensis* strain HB-2 and 99.71% with *B. altitudinis* strain MKBT. Lower similarity was recorded with other bacilli such as *B. pumilus*, *B. stratosphericus*, *B. aerophilus*, and *B. australimaris*. Therefore, the strain was given the name *B. xiamenensis* strain ASP-J1-4, with accession number PQ593941 (Table 1 and Figure 1).

### 2.2. Purification of L-ASNase from B. xiamenensis Isolate ASP-J1-4

**Figure 2:**
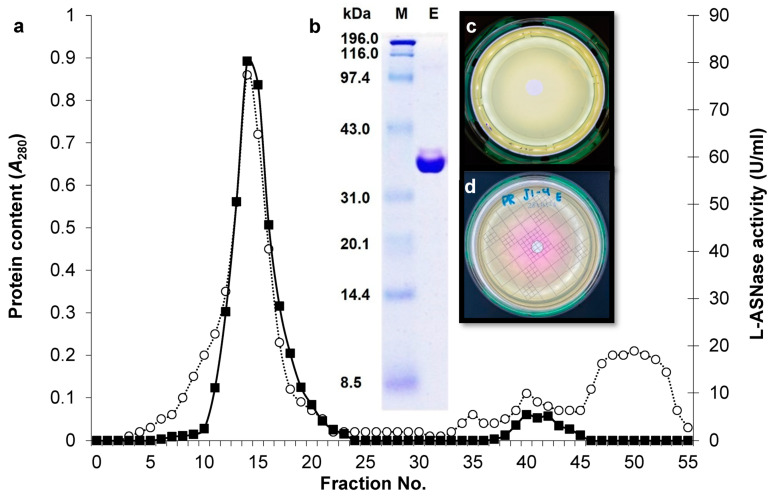
Elution profile of the purified L-ASNase from *B. xiamenensis* ASP-J1-4 (**a**). SDS-PAGE analysis confirms its purity at 36 kDa (**b**). Plate assays involving the specific substrates did not show L-GTNase activity (**c**) but showed high L-ASNase activity (**d**).

The eluted L-ASNase from strain ASP-J1-4 through DEAE-Sepharose ion exchanger (Figure 2a) showed a major monomeric band at 36 kDa (Figure 2b). Interestingly, the purified enzyme did not show any L-GTNase activity on glutamine plates compared with L-ASN plates (Figure 2c,d).

### 2.3. Effect of pH and Temperature on ASP-J1-4 L-ASNase Activity and Stability

**Figure 3:**
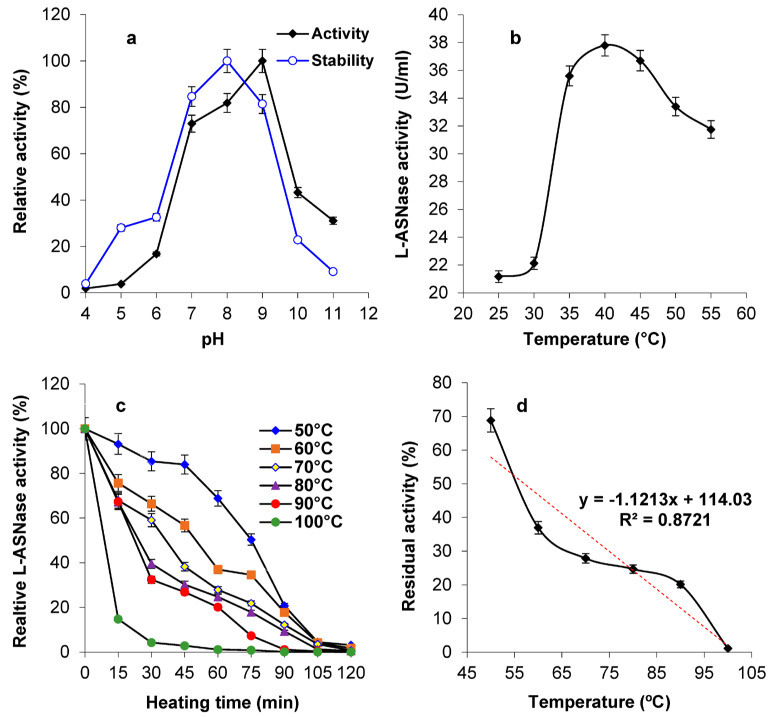
Impact of pH on enzymatic activity and stability (**a**). The impact of temperature on enzyme activity and stability is declared in panels (**b**,**c**), respectively. Panel (**d**) shows the deduced midpoint temperature (*T*_m_).

The enzyme showed optimal activity at pH 9.0 and stability at pH 7–9 (Figure 3a). In parallel, it exhibited optimal activity at 40 °C (Figure 3b), showing a half-life time of 24 min to 9 min at temperatures of 50 °C to 100 °C, respectively (Figure 3c). The final midpoint temperature was 57.1 °C (Figure 2d).

### 2.4. Effect of Metal Ions and Reagents on ASP-J1-4 L-ASNase Activity

**Figure 4:**
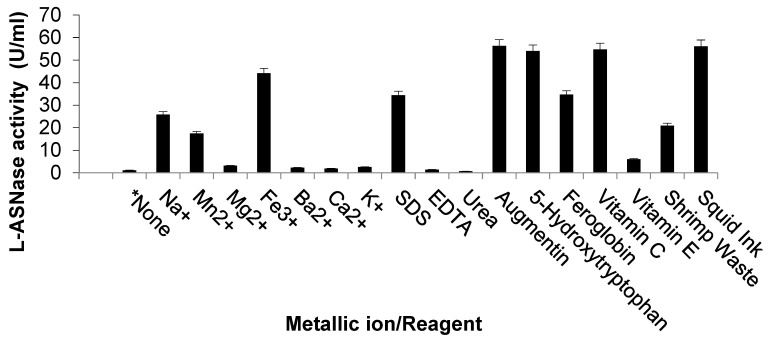
Effect of metallic ions in addition to L-ASNase reagents and supplements on enzymatic activity.

Tested metallic ions were stimulatory, with maximum induction in the case of Fe^3+^, Mn^2+^, and Na^+^, respectively (Figure 4). Most common reagents enhanced the activity of ASP-J1-4 enzyme; however, urea seemed to be an inhibitory agent, while EDTA did not show any impact on enzymatic activity (Figure 4).

### 2.5. Evaluation of ASP-J1-4 L-ASNase Kinetics

**Figure 5:**
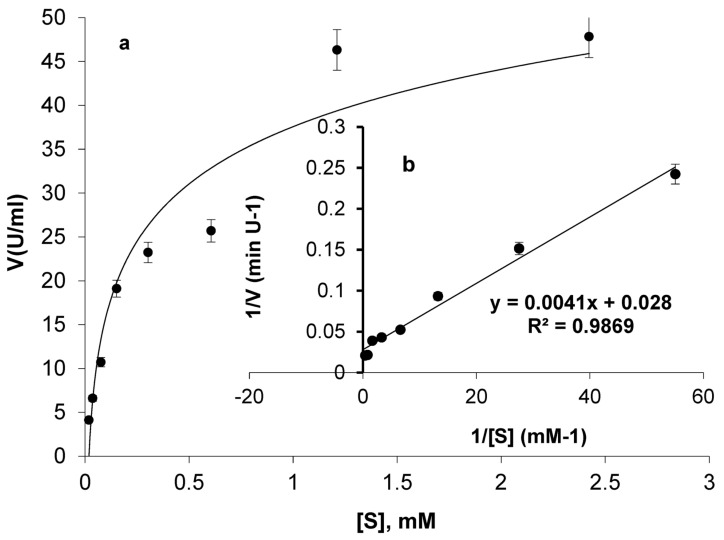
L-ASNase dynamics against different concentrations of L-ASN in the form of a hyperbolic plot (**a**) and Lineweaver–Burk plot (**b**).

The catalysis of ASP-J1-4 against L-ASN at concentrations 0.02 mM to 2.42 mM was plotted in the form of a hyperbolic curve and Lineweaver–Burk plot (Figure 5). A steady rise in catalysis was observed by increasing L-ASN concentration, followed by a small increase at higher concentrations. The enzyme showed a *V*_max_ of 35.71 U/mL and a *K*_m_ of 0.15 mM.

### 2.6. Antitumor Activity

#### 2.6.1. Dose-Dependent Reduction of HeLa and HCT-116 Cell Proliferation and Viability

**Figure 6:**
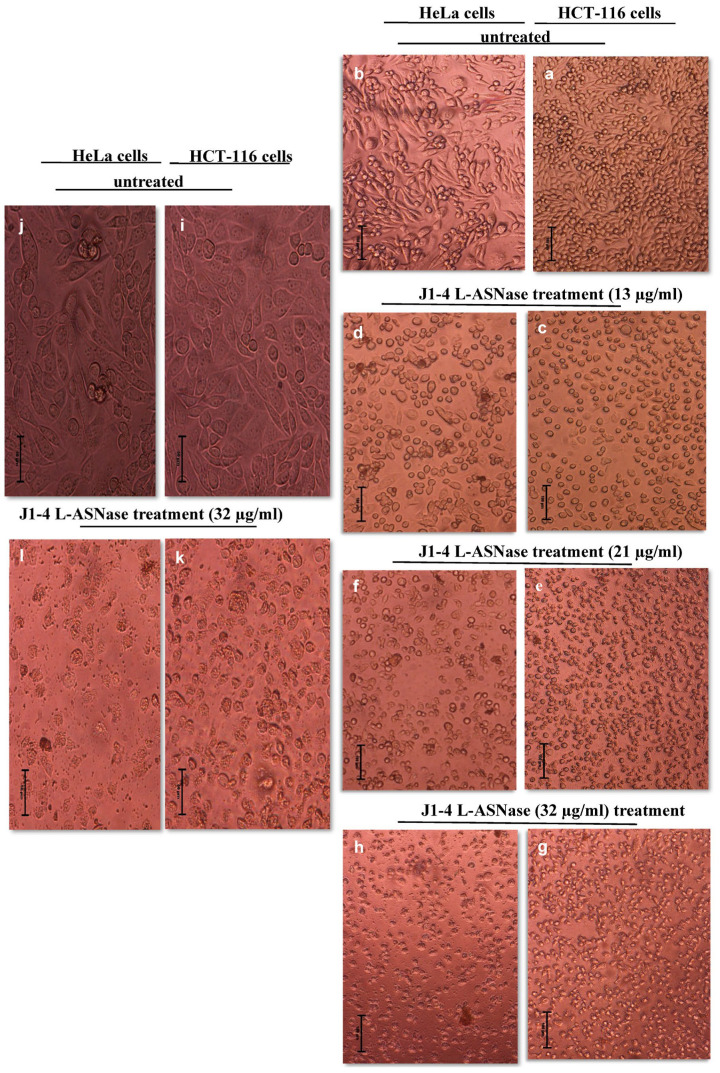
Microscopy-based cell proliferation data representing the effect of ASP-J1-4 treatment (13–32 µg/mL) on HCT-116 and HeLa cell viability. The right panels, (**a**,**c**,**e**,**g**,**i**,**k**), represent the HCT-116 tumor cell line; however, the left panels, (**b**,**d**,**f**,**h**,**j**,**l**), represent the HeLa tumor cell line. Cell magnification was under 20× (**a**–**h**); however, panels (**i**–**l**) represent 40× magnification of control cells and cells treated with 32 µg/mL of enzyme.

We have investigated whether ASP-J1-4 affected cell proliferation in HeLa and HCT-116 cells; the effects were assessed by cell proliferation and viability assay as stated in the Section 4. Compared to untreated control cells HCT-116 (Figure 6a,i) and HeLa cells (Figure 6b,j), treated HCT-116 cells with 13 µg/mL of enzyme for 24 h (Figure 6c) and treated HeLa cells (Figure 6d) show lower density and a rise in number of round and floating cells. HCT-116 cells treated with 21 µg/mL show a significant decrease in cell number, with the majority becoming round, floating, and losing their ability to divide. Similarly, HeLa cells showed a considerable decrease in cell density and a rise in rounded and shrunken cells (Figure 6f). Further, cells treated with 32 µg/mL of ASP-J1-4 showed a noticeable decline in cell health related to the untreated cells, a decrease in adhering cells, and a notable rise in the proportion of rounded and shrunken cells (Figure 6g,h,k,l).

#### 2.6.2. ASP-J1-4 L-ASNase Treatment-Induced Nucleus Morphological Changes

**Figure 7:**
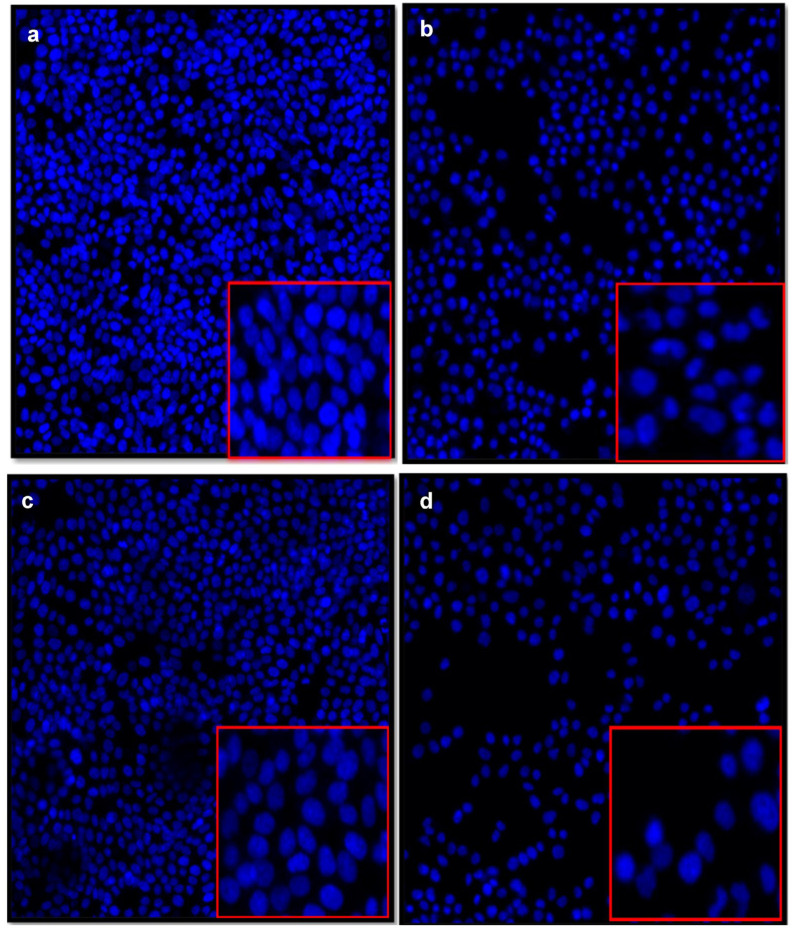
DAPI staining showing the nuclear morphology of HCT-116 and HeLa cells before (**a**,**c**) and after ASP-J1-4 treatment (**b**,**d**), respectively. Untreated HCT-116 (**a**) and HeLa cells (**c**) exhibit normal nuclear morphology with well-defined, round, and uniformly stained nuclei, indicating healthy, viable cells. However, cells treated with 32 µg/ml ASP-J1-4 for 24 h display characteristic apoptotic features (**b**,**d**). The nuclei show chromatin condensation, nuclear fragmentation, and shrinkage—all indicators of apoptosis. There were widespread abnormal nuclear shapes and dense chromatin bodies across the cell population, contrasting starkly with the control images. Images were captured using an Olympus BX51 fluorescence microscope (Olympus Corporation, Tokyo, Japan) at 20× magnification.

To investigate the impact of ASP-J1-4 on the viability of cancer cells, and to check whether the decline in survival rate was attributed to programmed cell death, DAPI staining was performed, and fluorescence microscopy was applied to verify the apoptotic bodies, which are characterized by their morphological alterations in their nuclei. Our data show that HCT-116 cells treated with 32 µg/mL of ASP-J1-4 demonstrated a reduction in cell number and nucleus morphological changes as an indicator of apoptosis (Figure 7b,d), which are particularly evident by characteristic nuclear condensation and fragmentation of apoptotic cells, related to the untreated control with no obvious signs of nuclear condensation or fragmentation, and the nuclei resemble a normal oval or round shape (Figure 7a,c).

#### 2.6.3. Viability of HCT-116 and HeLa Cells After Treatment with ASP-J1-4 and IC_50_ Calculation

**Figure 8:**
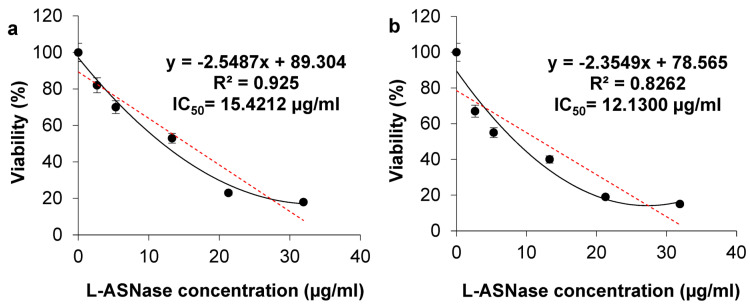
Dose–response curve of the cytotoxicity of ASP-J1-4 against HCT-116 (**a**) and HeLa tumor cell lines (**b**). The cells were exposed to the enzyme at various dosages for 24 h.

To investigate the cytotoxic effects of ASP-J1-4 upon HCT-116 and HeLa cells, the regularly used MTT assay was conducted, and cells were cultured with escalating ASP-J1-4 concentrations for 24 h (Figure 8). The results showed that both cell lines responded to ASP-J1-4 treatment with different degrees of sensitivity. As the MTT chart shows, L-ASNase treatment decreased HCT-116 and HeLa cell growth in a dose-dependent pattern. The untreated cells exhibited 100% viability and served as the baseline for the comparison.

## 3. Discussion

L-ASNases have industrial applications in minimizing the development of acrylamide carcinogens in food and treating cancer diseases by inhibiting the synthesis of proteins in tumor cells by depleting the availability of the amino acid L-ASN. Previous studies indicate that seawater is a major source of bacteria and fungi producing pharmaceutical enzymes [3,4]. Marine environments hold a unique bacterial diversity that could be used to produce enzymes with unique biochemical and biotechnological characteristics. Bacterial L-ASNases are categorized into two types based on cellular localization. Type I is a cytosolic and intracellular metabolite and has a lower affinity for L-ASN, while type II is periplasmic and extracellular and has high substrate affinity, such as the commercial L-ASNases of *E. chrysanthemi* (ErA) and *E. coli* (EcAII). Accordingly, the current research was directed to isolate local bacteria producing extracellular L-ASNases, which offer high amounts in the culture medium, ease of recovery without needing cell lysis, and can be purified economically [12].

In this research, the most potent isolate, *B. xiamenensis* ASP-J1-4, was recovered from *Suaeda maritima*, which is a halophyte that thrives in coastal areas with high salinity. It blooms from late summer to late fall, with seeds ripening in the fall along the coasts of the Eastern Province of Saudi Arabia in large numbers. To our knowledge, there are no previous reports on L-ASNase production from *B. xiamenensis*. The first isolation of this species was also from a marine source (Xiamen Island, China), where it was recovered from *Mugil cephalus* feces [13]. Recently, it was found to produce indole-3-carboxaldehyde, which inhibited the growth of the harmful bacterium *Moraxella osloensis* [14]. Additionally, we have isolated *B. altitudinis* isolate J1-2 from *Suaeda maritima*. The literature only described two studies on L-ASNase production from this species; Lailaja et al. [15] isolated marine *B. altitudinis* isolate 105 from crabs, and its enzyme was extracellularly produced and lacked concurrent L-GTNase activity, as confirmed by our findings. Hadi et al. [16] recovered the second isolate *B. altitudinis* KB1 from a sea beach in Kerala, India.

From a shoreline soil sample and sponge #1, we have recovered J3-1 and SPBR-6 isolates, respectively; both were identified as *B. velezensis*. Hozoorbakhsh et al. [17] have also isolated an L-ASNase-producing strain from effluents of the Isfahan slaughterhouse, Iran. Mostafa et al. [12] also isolated a marine strain of *B. velezensis* producing L-GTNase-free L-ASNase from sediment samples of the Red Sea, Saudi Arabia. From mangrove sediment #1, we have isolated *Priestia flexa* MR-25, which was formerly known as *B. flexus*. Also, Chand et al. [18] isolated *B. flexus* strain SS, producing the same enzyme without any L-GTNase activity. We have also recovered isolate MR-7 from the same sediment, and it was identified as *B. licheniformis*. L-ASNase production from this species is common. For example, a *B. licheniformis* isolate (accession number MG665995) was recovered from a soil sample with the ability to produce type I and type II L-ASNases [19]. A marine isolate designated as KKU-KH14 was recovered from the Red Sea of Saudi Arabia. The L-GTNase activity was not detected in the ASNase preparations [11]. However, the soil isolate *B. licheniformis* RAM-8 showed a combined L-GTNase activity of 0.8 IU/mL [20]. From mangrove sediment #2 and sea star #2, we have recovered isolates MQ1-1 and STH-5, respectively; both were identified as *B. subtilis*, which is a common producer of L-ASNase. For example, type II L-ASNase from a halotolerant *B. subtilis* CH11 was recovered from *Chilca salterns* in Peru and was cloned in *E. coli* BL21 (DE3) pLysS [21]. Fortunately, our extensive screening program introduced many other L-ASNase producers for the first time, such as *B. paralicheniformis* HOR-5 and SPF-4, *Paenibacillus lautus* J6-1, *Staphylococcus epidermidis* HOR-4, *Proteus mirabilis* A-2, *M. caseolyticus* K-3, *Psychrobacter sanguinis* K2-2, and *Psychrobacter phenylpyruvicus* STF-1 (Table 1).

The purified enzyme from ASP-J1-4 by DEAE-Sepharose ion exchanger showed a major monomeric band at 36 kDa without any L-GTNase activity. This molecular mass resembles L-ASNases of *B. licheniformis* KKU-KH14 [11], *Pseudomonas* sp. PCH199 [22], and the commercial L-ASNases of *E. chrysanthemi* 3937 and *E. coli* [23]. The molecular mass of L-ASNase of the marine *B. velezensis* was 39.7 kDa [12]; however, *B. flexus* SS enzyme showed 33 kDa [18]. Other bacterial sources also showed 33–37 kDa, such as *Vibrio cholerae* [24], *B. altitudinis* [25], *Ps. aeruginosa* [26], *Pseudomonas* sp. PCH199, and *Pseudomonas* sp. PCH44 [27]. However, the recombinant enzyme of *B. subtilis* CH11 showed a homotetrameric form at 155 kDa [21].

The current enzyme showed optimal activity at pH 9.0 and stability at pH 7–9, predicting its functioning inside the human body. Also, pH 9.0 exerted maximum catalytic activity for those from *B. subtilis* CH11 [21] and *B. licheniformis* [28]. The current values are also close to other studies; *B. altitudinis* strain 105 L-ASNase displayed optimal stability and activity at pH 7–8 [15]. L-ASNase from marine *B. velezensis* was maximally active at pH 7.5 and maximally stable at pH 8.5 [12]. L-ASNases from *B. subtilis* showed maximum activity at pH 7.5 [29] and pH 5.0 [30]. pH 8.0 was best for L-ASNases of *B. licheniformis* [19]. The extremozyme of *Pseudomonas* sp. PCH199 showed maximum performance at pH 8.5 [22]. The L-ASNase from *B. licheniformis* (accession number MG665995) exhibited optimum activity at pH 8.6 [19]. The maximum activity and stability of *B. licheniformis* KKU-KH14 enzyme was at pH 7.5 and 8.5, respectively [11]. It is evident that alkaline pHs are optimal for L-ASNases. This may be attributed to the low affinity of the end product aspartic acid to bind the active moiety, which allows a high affinity of substrate molecules to react with the enzyme. In addition, at low pH values, the released aspartic acid has a great affinity to bind enzyme molecules, which hinders the binding of L-ASN [12].

In parallel, the ASP-J1-4 enzyme exhibited optimal activity at 40 °C, showing a half-life time of 24 min to 9 min at temperatures from 50 °C to 100 °C, respectively. The final midpoint temperature was 57.1 °C, predicting the functioning inside the human body. These results agree with the extracellular L-ASNases from *B. licheniformis* (40 °C, [19]), *B. subtilis* (40 °C, [29]), *B. subtilis* (37 °C, [30]), *B. altitudinis* strain 105 (37 °C, [15]), *B. velezensis* (37 °C, [12]), and *B. licheniformis* KKU-KH14 (37 °C, [11]). However, our optimal temperature is different from the N-truncated L-ASNases of *Yersinia pseudotuberculosis* (60 °C, [31]), *B. amyloliquefaciens* MKSE (65 °C, [32]), *B. subtilis* WB600 (65 °C, [33]), and *Pseudomonas* sp. PCH199 (60 °C, [22]). Other L-ASNases showed less stability at 50 °C, such as those from *Enterobacter cloacae* [34], *Paenibacillus barengoltzii* [35], *V. cholerae* [24], and *B. aryabhattai* ITBHU02 [36]. The significance of thermostability of ASP-J1-4 is that it imparts cost-effectiveness to enzyme in terms of prolonged transportation and storage shelf-life. The thermostability of L-ASNase in the present study is unsurprising and is consistent with adaptive features of microorganisms harboring in arid and semiarid environments such as the Gulf region [4].

Tested metallic ions were stimulatory, with maximum induction in the case of Fe^3+^, Mn^2+^, and Na^+^, respectively. Enzymatic activation by iron was also reported by other researchers [22]. The activity of the recombinant L-ASNase from *B. subtilis* CH11 was considerably enhanced by K^+^, Ca^2+^, and Mg^2+^ ions [21]. However, some authors reported an inhibitory effect of Mn^2+^ ions [30,33]. The activity of *Pseudomonas* sp. PCH199 extremozyme decreased to 51.6% by K^+^ and was enhanced by 127.5% and 111.6% in the presence of Na^+^ and Ca^2+^, respectively [22]. The activation of enzymes by some metal ions may be due to their ability to act as cofactors for binding at the catalytic site of the enzyme, while the suppression by other ions may be attributable to their chelation with sulfhydryl groups of protein structures [4,27].

The stimulative effect of NaCl on ASP-J1-4 activity is similar to that from *B. megaterium* strain MG1 [37] and *B. megaterium* H-1 [38]. Also, the activity of L-ASNase from *B. licheniformis* was enhanced by 48.0% in the presence of 500 mM NaCl [19]. Water molecules have a substantial impact on the functionality of enzymes, as they are imbibed in the outer and inner parts of their structures when water activity is affected by extreme salinity, temperature, and pH, because water is considered as the main weak point in enzyme inhibition. Stimulation of enzymes by NaCl may be attributed to its ability to induce loop flexibility in the integrity of halophilic enzymes. Such enzymes have high negative charges, so they can be easily dissociated and become more flexible in the presence of NaCl [19].

Most commonly used reagents in the study enhanced the activity of ASP-J1-4 enzyme; however, urea seemed to be an inhibitory agent, while EDTA did not show any impact on enzymatic activity. Similarly, EDTA did not affect L-ASNases of *Pseudomonas* sp. PCH199 [22], *B. megaterium* strain MG1 [37], and *B. megaterium* H-1 [38]. In addition, EDTA and SDS did not influence the catalysis of the extremozyme from *Pseudomonas* sp. PCH199 [22]. This was attributed to the lack of serine residue in the building of the later L-ASNases. However, the activity of *B. subtilis* CH11 enzyme was slightly inhibited by EDTA and completely inhibited by SDS [21]. The suppressive effect of reducing agents might be attributed to their ability to decrease protein aggregation by decreasing the intermolecular disulfide bridge formation. This was observed for L-ASNases from *Pectobacterium carotovorum* [39] and *E. carotovora* [40].

Therapeutic adjuncts to commercial L-ASNases such as antibiotics, 5-hydroxytryptophan, feroglobin, vitamin C, vitamin E, shrimp waste, and squid ink during treatment regimens were found to exert a stimulatory effect towards ASP-J1-4 enzyme. This supports the idea that the catalytic performance of therapeutic enzymes can change when administered by patients [21]. For example, activity of the recombinant ScASNase1 L-ASNase from *Saccharomyces cerevisiae* increased two-fold when mixed with human serum [41]. Additionally, the catalysis of *E. chrysanthemi* L-ASNase was stimulated when mixed with osmolytes [42]. Therefore, supplement assays for the efficacy of these enzymes in the presence of reagents are important to assess their medical application.

A steady rise in catalysis was observed by increasing L-ASN concentration, ended by a small increase at higher concentrations. This may be due to the saturation of active moieties by substrate molecules [1]. The enzyme showed a *V*_max_ of 35.71 U/mL and a *K*_m_ of 0.15 mM. This coincides with the *K*_m_ of enzyme from *Pseudomonas* sp. PCH199 (0.16 mM). However, in the latter, L-GTNase activity was detected with a *K*_m_ of 0.034 mM [22]. In parallel, lower values of *K*_m_ were reported for L-ASNases from *B. altitudinis* 105 (0.02 mM; [12]), *B. velezensis* (0.04 mM; [15]), *E. coli* (0.02–0.05 mM, [43]), *E. chrysanthemi* (0.05 mM, [41]), and the commercially existing *E. coli* (0.01 mM). However, various studies reported higher values of *K*_m_ for L-ASNases from *Ps. oryzihabitans* (10 mM, [44]), *B. altitudinis* (90.9 mM, [25]), *B. halotolerans* (4.7 mM, [45]), *B. amyloliquefaciens* (1.5 mM, [32]), *Paenibacillus barengoltzii* (3.6 mM, [35]), *B. licheniformis* KKU-KH14 (50 mM, [11]), *B. subtilis* CH11 (4.75 mM, [21]), *B. subtilis* WB600 (5.29 mM, [33]), *B. subtilis* (0.43 mM, [29]), and *B. licheniformis* (40 mM, [19]).

Kinetic constants and the dynamics of enzymes are crucial for commercialization. The in vitro studies do not always express their catalytic efficiency upon release in human blood. For example, the activity of ScASNase1 increased twofold when mixed with human serum [41]. Furthermore, L-ASNase from *E. chrysanthemi* increased its *V*_max_ in the presence of osmolytes [42]. The lower value of *K*_m_ serves as proof of the affinity and dynamics to substrate degradation and indicates high substrate specificity, which is crucial in therapeutic applications [1,2]. L-ASN concentration in normal serum is around 50 μM. Thus, ASP-J1-4 could be a promising therapeutic L-ASNase with high catalytic efficiency and stability at different temperatures and pH levels.

HCT-116 and HeLa cells, treated with 13 µg/mL of enzyme, showed lower density and a rise in the number of round and floating cells, suggesting a growth-inhibiting impact and a toxic impact at lower concentrations. In addition, HCT-116 cells treated with 21 µg/mL show a significant decrease in cell number, with the majority becoming round, floating, and losing their ability to divide, indicating a high rate of cell death and a toxic impact of L-ASNase at a 21 µg/mL dose. Similarly, HeLa cells showed a considerable decrease in cell density, and a rise in rounded and shrunken cells, suggesting that the enzyme’s action is more noticeable at this dose. Furthermore, cells treated with 32 µg/mL of ASP-J1-4 showed a noticeable decline in cell health related to the untreated cells, a decrease in adhering cells, and a notable rise in the proportion of rounded and shrunken cells, suggesting a higher rate of apoptosis. The decreased cell viability and altered nuclear morphology show that ASP-J1-4 effectively induces apoptosis in HCT-116 and HeLa cell lines. Tumor cells treated with L-ASNases of *Zymomonas mobilis* [46] and *Enterobacter cloacae* [34] showed morphological changes in their nuclei due to apoptotic cell death after DAPI staining, the same as ASP-J1-4 enzyme.

The calculated IC_50_ values were 15.42 µg/mL and 12.13 µg/mL, respectively, which suggests that the cells are highly sensitive to the enzyme, especially HeLa cells, as evidenced by the low IC_50_ value. These values are comparable to the L-ASNase from *B. velezensis* toward the breast adenocarcinoma cell lines MDA-MB-231 (IC_50_ 12.6 μg/mL) and MCF-7 (IC_50_17.3 μg/mL) [12], the L-ASNase from *B. licheniformis* KKU-KH14 toward MCF-7 (IC_50_ 14.55 µg/mL), HepG-2 (IC_50_ 11.66 µg/mL), and HCT-116 cell lines (IC_50_ 17.02 µg/mL), and the L-ASNase from *B. flexus* SS against the WIL2-S tumor cell line (IC_50_ 16.2 µg/mL) [18]. However, the later L-ASNase from *B. flexus* SS showed a lower IC_50_ value towards the tumor cell line SKBR3 (IC_50_ 0.8 µg/mL) and higher IC_50_ value against TF-1 (IC_50_ 47 µg/mL) [18].

The lack of cytotoxic effect in the absence of L-ASNase may be due to the activity of L-ASN synthetase that utilizes the substrate from other processes during starvation. The lower the IC_50_ value, the more efficacious the drug is at low doses, and, consequently, the lower the systemic toxicity when administered by humans. The IC_50_ value of ASP-J1-4 is relatively low, suggesting a promising drug with a lower dosage. Although the present study utilized validated and complementary assays (Trypan blue, DAPI, and MTT) to assess the cytotoxic effect of ASP-J1-4, future studies may include soft agar colony formation, flow cytometry, or fluorescence-based live/dead assays to further characterize the enzyme’s impact on long-term clonogenic potential, cell cycle progression, and apoptosis mechanisms in different cancer cell lines.

## 4. Materials and Methods

### 4.1. Materials and Reagents

L-ASN, DEAE-Sepharose, and SDS-PAGE marker proteins were obtained from Merck (Rahway, NJ, USA). Other substances were of analytical grade and sourced from local suppliers. HCT-116 and HeLa tumor cell lines were obtained from the Institute for Research and Medical Consultations (IRMC) of Imam Abdulrahman Bin Faisal (IAU) University, Saudi Arabia. Nessler’s reagent was freshly made by combining 2.2 g of HgCl_2_ with 45 mL of distilled water (dH_2_O) and stirring till complete dissolution (Solution A). In parallel, 6.0 g of KI was dissolved in the lowest amount of dH_2_O (Solution B), and 7.5 g of KOH was dissolved in 25 mL of dH_2_O (Solution C). Solution B was added dropwise to solution A followed by the addition of solution C. Finally, the total volume was adjusted to 100 mL and stored in an airtight, amber-glass bottle to prevent photodegradation [38]. 

All instruments used during this study were specified with their model and manufacturer to ensure reproducibility. L-ASNase activity was quantified using a UV-VIS spectrophotometer (SHIMADZU UV-1800, SHIMADZU Corporation, Kyoto, Japan). Fluorescence microscopy for DAPI-stained nuclei was performed using an Olympus BX51 microscope (Olympus Corporation, Tokyo, Japan). SDS-PAGE electrophoresis was carried out with a Mini-PROTEAN^®^ Tetra Cell System (Bio-Rad, Hercules, CA, USA). For enzyme purification, an NGC™ Chromatography System (Bio-Rad, Hercules, CA, USA) was used. These instruments were operated according to manufacturer instructions under standard laboratory conditions.

### 4.2. Sample Collection and Isolation of l-ASNase-Producing Bacteria

Marine plants, algae, animals, and sediments were collected from the Arabian–Persian Gulf shoreline of Saudi Arabia after obtaining permission and approval from IAU University and relevant authorities. The samples were promptly moved to the laboratory in sterile polythene bags. The marine animals were kept in glass aquaria under optimum condition for 24 h. Their waste in the surrounding water was used for bacterial isolation. These were then returned to their habitats by qualified divers. To isolate endophytic bacteria, marine plants and algae were sterilized exteriorly by soaking in 0.5% (*w*/*v*) sodium hypochlorite for 2 min, then rinsed with sterile dH_2_O under aseptic condition. They were then transferred to a sterilized mortar and ground thoroughly. Finally, each 1 mL homogenate or 1 g sediment was suspended in 9 mL of 0.84% NaCl and serially diluted till 10^−3^ dilution. Then, 100 µL of each was disseminated onto M9 agar consisting of (g/L) glucose 7, L-ASN 10, nutrient broth 8, NaCl 15, phenol red 0.009, and agar 15. The final pH was adjusted at 7.0, and incubation was performed at 37 °C for 48 h. L-ASNase-positive microorganisms were selected by the appearance of pink halos around their growing colonies (qualitative assay) [12].

### 4.3. Identification of Potential Isolates

The top bacterial candidates were identified using *16SrDNA* gene sequencing, and a phylogenetic tree was built using the neighbor-joining approach for species-level confirmation. During this process, the genomic DNA was extracted, and amplification of the target gene was performed using the universal primers 16SF (27F 5′-AGAGTTTGATCMTGGCTCAG-3′) and 16SR (1492R 5′-TACGGYTACCTTGTTACGACTT-3′). The PCR reaction was executed in a total volume of 25 μL, containing 1× Taq buffer, 200 μM dNTPs (MBI Fermentas, Amherst, NY, USA), 0.5 μM each of forward and reverse primers, 1.0 mM MgCl_2_, 1.25 U Taq DNA polymerase (MBI Fermentas, Amherst, NY, USA), and 1 μL of template DNA. The cycling program involved DNA denaturation at 98 °C for 3 min, 30 cycles of denaturation at 94 °C for 1 min, annealing at 53 °C for 1 min, and extension at 72 °C for 10 min. Amplified products of the expected size were purified using the QIAquick PCR purification kit (Qiagen, Hilden, Germany) and sequenced with an ABI PRISM 377 genetic analyzer (Applied Biosystems, Foster City, CA, USA) with 907R (5′-CCGTCAATTCMTTTRAGTTT-3′) and 785F (5′-GGATTAGATACCCTGGTA-3′) primers. The obtained sequences were compared with those deposited in the National Center for Biotechnology Information (NCBI) database using the BLASTN option. For phylogenetic assessment of the potent L-ASNase-producing strain ASP-J1-4, multiple sequence alignment was conducted using the CLUSTALW program, aligning the obtained sequence with GenBank database entries. The following strains with corresponding accession numbers were used in the construction of the tree: *Bacillus* sp. strain RPS04 (PQ346053), *B. xiamenensis* strain HB-2 (OR342733), *B. altitudinis* strain MKBT (MH169240), *B. aerius* strain SUF02NA (MT052654), *B. pumilus* strain SU18-05 (MH201274), *B. stratosphericus* strain T-7.3 (OR733705), *B. aerophilus* strain OdE19 (OR964106), *B. pumilus* strain G-06 (KY970107), *B. australimaris* strain CGK221 (OM658338), and *B. altitudinis* strain L5 (MN134575). Sequence alignment and phylogenetic tree construction were conducted using the *build* function of ETE3 version 3.1.3 via GenomeNet (https://www.genome.jp/tools/ete/, accessed on 27 March 2025), applying 1000 bootstrap replicates to assess reliability.

### 4.4. Production and Quantitative Assay of L-ASNase

The crude enzymes were prepared by cultivation of bacteria in M9 basal medium. The cultures were incubated for 48 h under the optimized conditions. L-ASNase activity was assessed after separation of cells by centrifugation for 20 min at 5000 rpm. For L-ASNase activity measurement, 0.1 mL of enzyme was combined with 1 mL of 100 mM Tris-HCl buffer (pH 7.0) containing 10 mM L-ASN. The reaction was carried out for 30 min at 37 °C and was stopped by the addition of 0.5 mL 100 mM trichloroacetic acid. Precipitated proteins were separated through centrifugation, and supernatant was mixed with 0.2 mL of 100 mM EDTA and 1 mL of 1 N NaOH. An equivalent volume of dH_2_O was used in place of enzyme for the control reaction. The liberated ammonia was quantified by adding 0.5 mL of Nessler’s reagent to the reaction mixture. The yellow-to-brown color due to the hydrolysis of L-ASN was quantified by a UV-VIS spectrophotometer (SHIMADZU) at *A*_436_ after 5 min of incubation. Each unit (U) of enzyme activity was identified as the amount of L-ASNase that releases one µmol of ammonia in one min at 37 °C under the standard conditions of reaction. For this purpose, a calibration curve was generated using solutions with defined concentrations of ammonium sulfate in the range of 0–200 mg/mL, then the corresponding absorbances were measured. For L-GTNase activity measurement, the same procedure was applied; however, L-ASN was replaced with L-glutamine. The most potent isolates from these assays were preserved for long-run uses in 15% (*v*/*v*) glycerol at −80 °C. For short-run uses, these were subcultured on agar media and preserved at 4 °C.

### 4.5. Purification of L-ASNase

This was performed through multiple stages comprising acetone precipitation, dialysis, and ion exchange chromatography. Fermentation broth was separated from the bacterial cells by centrifugation at 5000 rpm for 20 min (HETTICH Centrifuge RIF 1406, Raptor Supplies, Manchester, UK), then soluble proteins were salted out by gradual addition of cold acetone with vigorous stirring till a 60% (*v*/*v*) concentration limit. The enzyme was dialyzed to remove the nonprotein structures. The dialysis tube was put in 250 mL of 500 mM chilled sodium borate buffer (pH 8.6) with stirring at 4 °C for 12 h. The dialyzed free proteins were eluted at 1 mL/min through a DEAE-Sepharose column (1 × 15 cm) pre-equilibrated using 50 mM Tris-HCl buffer (pH 7.0, buffer A). The NGC system (Bio-Rad, USA) was used in the separation process. However, the bounded proteins were eluted with 50 mM Tris-HCl buffer (pH 8.5) containing 0.42% (*v*/*v*) acetone with a linear rise in NaCl strength from 20 mM to 800 mM (buffer B). Fractions were collected based on absorbance readings at 280 nm and evaluated for enzymatic activity. Active fractions were pooled, lyophilized, and kept at 4 °C for characterization studies. SDS-PAGE was performed to estimate the enzyme’s molecular mass and homogeneity, utilizing 4% stacking gel and 12% resolving gel at a constant voltage of 200 V. Protein bands were stained by immersion in Page Blue for 5 min and destained by soaking in dH_2_O for another 5 min.

### 4.6. Characterization of L-ASNase

#### 4.6.1. Impact of Temperature and pH on Enzyme Stability and Activity

The impact of pH was examined between 4.0 and 11.0 using 20 mM sodium acetate buffer (pH 4.0–6.0), 20 mM sodium phosphate buffer (pH 6.5–8.0), and 20 mM glycine–NaOH buffer (pH 8.5–11.0) with an incubation of 30 min. Stability of L-ASNase was assessed by preincubation at various pH levels for 1 h, after which the remaining activity against L-ASN was measured using 20 mM phosphate buffer at pH 8.0. To determine the enzyme’s optimal temperature, the enzyme–substrate combinations were incubated for 30 min across 25–55 °C at pH 8.0, and the amount of ammonia released was quantified using Nessler’s method. For evaluating stability, the enzyme was preincubated at 50–100 °C for varying periods (15–120 min) before activity assessment. Residual activity was then measured against 10 mM of L-ASN with 20 mM sodium phosphate buffer (pH 8.0) at 40 °C. The half-life time (*T*_1/2_) and the midpoint temperature (*T*_m_) were then calculated [1].

#### 4.6.2. Effect of Metal Ions and Reagents on Enzyme Activity

Understanding how an enzyme interacts with various chemicals that may impact its catalytic performance was the goal of this experiment. The effect of common reagents such as ethylenediaminetetraacetic acid (EDTA) (2 mM), urea (2.5 mM), and sodium dodecyl sulphate (SDS) (2 %) on enzymatic activity was investigated. In addition, the effect of co-supplements to therapeutic L-ASNases such as Augmentin antibiotic, 5-hydroxytryptophan, feroglobin, ascorbic acid (vitamin C), tocopherol (vitamin E), shrimp waste, and squid ink was tested at a concentration of 1% (*w*/*v*). Furthermore, the effect of MgCl_2_, CaCl_2_, NaCl, KCl, MnCl_2_, FeCl_3_, and BaCl_2_ metallic ions on the activity of the enzyme was investigated at a final concentration of 1 mM. For all, the remaining activity against L-ASN was assessed after preincubation of enzyme for 30 min at 40 °C with respective inhibitor and/or metal ions.

#### 4.6.3. Determination of Kinetic Constants

The dynamics of L-ASNase against concentrations of 0.02 mM to 2.42 mM of L-ASN were tested. Activity was determined under the regular conditions, then the maximum velocity of reaction (*V*_max_) and the Michaelis–Menten constant (*K*_m_) were evaluated from the Lineweaver–Burk curve equation.

### 4.7. Antitumor Activity

#### 4.7.1. Cell Culture of Human Cell Lines

The HCT-116 tumor cells were cultivated in Roswell Park Memorial Institute (RPMI-1640) medium augmented with 100 μg/mL streptomycin, 100 μg/mL penicillin, and 10% fetal bovine serum. However, the immortalized human HeLa cells were cultivated in Dulbecco’s Modified Eagles medium (DMEM) augmented with 4.5 g/L glucose, 100 µg/mL penicillin, 100 µg/mL streptomycin, and 10% newborn calf serum. Incubation of both cell types was performed at 37 °C in a humidified incubator with a 5% CO_2_ atmosphere (Thermo Fisher Scientific Inc., Waltham, MA, USA). Cells were passaged after trypsinization with trypsin/EDTA and subsequently neutralized by the addition of culture. Later, cells were diluted and plated into cell culture plates for further use.

#### 4.7.2. Cell Proliferation and Cell Viability Assessment

The proliferation and viability of HCT-116 and HeLa cells were determined by the trypan blue dye test. Cells were suspended in a 6-well plate and grown to the required density for 24 h. They were then treated with 13–32 µg/mL of L-ASNase and incubated for another 24 h. Trypan blue staining was applied to assess the viable cell count by mixing 10 µL of cell suspension with an equivalent volume of trypan blue. Exactly 10 µL of the mixture was added onto a hemocytometer or a cell counting slide, and the living cells were counted under a microscope (Countess ^TM^ II Automated Cell Counter, Thermo Fisher Scientific, Waltham, MA, USA). Dead cells are specifically stained by trypan blue, making it possible to distinguish between living and nonliving cells.

#### 4.7.3. Nuclear Morphology Evaluation Using DAPI Staining and Fluorescent Microscopy

DAPI staining is a qualitative assay, as it primarily provides information on the morphological changes in cell nuclei (such as in apoptosis). Alterations in the nuclear morphology of cells after exposure to ASP-J1-4 L-ASNase were evaluated by staining HCT-116 and HeLa cells with 4′,6-diamidino-2-phenylindole dihydrochloride (DAPI) stain. Cells were seeded in a 96-well plate with 32 µg/mL of tested enzyme and incubated at 37 °C in 5.0% CO_2_ for 24 h. Next, the treated cells were fixed using paraformaldehyde for 30 min, and then physiological buffered saline (PBS) was used to wash cells. DAPI staining was used to stain the cells for 15–20 min, and fluorescence microscopy using a MIC-01 Carl-Zeiss fluorescence microscope (ZEISS Spectroscopy, Jena, Germany) was used to examine the stained cell nucleus. 

#### 4.7.4. Cytotoxicity and Anti-Proliferative Determination of ASP-J1-4 L-ASNase on HCT-116 and HeLa Using MTT Assay

MTT assay is a quantitative assay. It is widely used to assess cell viability and proliferation. The MTT assay measures the metabolic activity of cells by assessing the reduction of MTT (a yellow compound) to formazan (a purple product) in viable cells. The amount of formazan formed is directly proportional to the number of viable cells, and it can be quantified by measuring absorbance. The cytotoxicity of different concentrations of ASP-J1-4 was evaluated using the universal 3-[4,5-dimethylthiazol-2-yl]-2,5 diphenyl tetrazolium bromide (MTT) assay. To determine the corresponding IC_50_ value, each cell line was treated separately with increasing concentrations of ASP-J1-4 for 24 h. Cell proliferation and viability of untreated (control) and treated HCT-116 and HeLa cells were determined at various time points. To standardize the cell density across various wells for the MTT experiment, the following equation was applied to measure cell proliferation or cytotoxicity [22]:

*Cell concentration per* mL = *(Number of cells counted/Volume of sample in* mL*) × Dilution factor*

Cells were suspended in 96-well plates at a concentration of 2 × 10^4^ cells per well and treated with several concentrations of ASP-J1-4 (13–32 µg/mL) and incubated at 37 °C in 5.0% CO_2_ for 24 h and 48 h. Dimethyl sulfoxide (DMSO) was used as a positive control, while untreated cells were used as a negative control. After incubation, the MTT solution was added to each well. The medium was then removed, and 200 µL of DMSO was replaced to solubilize the formazan crystals. Then, absorbances at wavelength 570 nm were measured using a microplate reader (BioTek Synergy H1 Multi-Mode Microplate Reader (Agilent Technologies, Santa Clara, CA, USA).

### 4.8. Statistical Analysis

Unless otherwise mentioned, the readings throughout this research were statistically analyzed using Microsoft Excel 365 to evaluate enzyme activity. Data were presented as averages of three technical replicates ± standard deviations.

## 5. Conclusions

L-ASNases have industrial applications in medicine and food. In this research, a promising L-ASNase-producing bacterium was isolated from a marine source and characterized as *B. xiamenensis* ASP-J1-4. It was able to produce high levels of the extracellular type. In addition, the biochemical characterization of the enzyme revealed high activity and stability under fluctuating temperatures and pH levels. Giving extra unique features such as high substrate-specificity, high stability in human physiological conditions, low *K*_m_, low IC_50_ dosage against HCT-116 and HeLa cancer cell lines, and the ability to trigger apoptosis makes it a promising bioproduct in the pharmaceutical industry. In vivo cytotoxicity validation and its efficacy determination take it a step further to establish this preparation as a medication for ALL. The study showed that the Arabian–Persian Gulf is a promising biological reservoir, as shown by the introduction of the new producer candidate of *B. xiamenensis*, which produces a L-GTNase-free L-ASNase that may be considered as a potential candidate for further pharmaceutical use as an anticancer drug.

## Figures and Tables

**Table 1:** Characterization of the most promising L-ASNase producers recovered from the marine environment of Saudi Arabia.

Isolate Id.	Accession No.	Source	Coordinate’s Location	L-ASNase Activity (U/mL)
*B. altitudinis* J1-2	PQ593940	Seablite *Suaeda maritima*	4HCC+FMC Al Jubail	49.07 ± 3.26
*B. xiamenensis* ASP-J1-4	PQ593941			53.20 ± 2.31
*B. velezensis* J3-1	PQ593943	Shoreline soil	4HCC+FMC Al Jubail	12.26 ± 0.85
*Paenibacillus lautus* J6-1	PQ593936			13.06 ± 0.76
*Psychrobacter phenylpyruvicus* STF-1	PQ593945	Seastar *Asteroidea* #1	C5V3+H4X Dammam	38.49 ± 2.24
*Proteus mirabilis* A-2	PQ593938	Marine alga *Eucheuma*	26.3249619, 50.2285868	31.34 ± 2.19 *
*Psychrobacter sanguinis* K2-2	PQ593953	Sea squids	26.182416, 50.165275	5.09 ± 0.22
*Macrococcus caseolyticum* K-3	PQ593933	*Planaria*	66QC+8PAL Khobar	8.79 ± 0.54
*B. velezensis* SPBR-6	PQ593947	Sponge #1	4XHV+FR6, unnamed road, Dhahran 34814	11.54 ± 0.85 *
*B. paralicheniformis* SPF-4	PQ593949	Sponge #2	GH74+HP5Khafji	1.65 ± 0.09
*B. subtilis* STH-5	PQ593951	Seastar *Asteroidea* #2	4XHV+FR6, unnamed road, Dhahran 34814	2.47 ± 0.08
*Priestia flexa* MR-25	PQ593935	Mangrove sediment #1	PXMV+Q7Ras Tanura	24.74 ± 1.15
*B. licheniformis* MR-7	PQ593957			5.64 ± 0.33
*B. subtilis* MQ1-1	PQ593959	Mangrove sediment #2	26.590850, 50.044333	3.16 ± 0.12
*Staphylococcus epidermidis* HOR-4	PQ593965	Seahorse *Hippocampus*	4XHV+FR6, unnamed road, Dhahran 34814	8.25 ± 0.43
*B. paralicheniformis* HOR-5	PQ593967			9.62 ± 0.45

* L-GTNase activity was detected; the values 1.24 U/mL and 48.52 U/mL were reported for strain A-2 and strain SPBR-6, respectively.

## Data Availability

The datasets in this research can be found in online repositories. The names of the repositories and accession numbers are included in this article.

## Abbreviations

The following abbreviations are used in this manuscript:

ALL	Acute type of Lymphoblastic Leukemia
DAPI	4′,6-diamidino-2-phenylindole dihydrochloride
DEAE	Diethylaminoethyl
DMEM	Dulbecco’s Modified Eagles medium
DMSO	Dimethyl sulfoxide
EDTA	Ethylenediaminetetraacetic acid
HCT	Human colon carcinoma
HeLa	Henrietta Lacks carcinoma
IC_50_	Half-maximal inhibitory concentration
*K*_m_	Michaelis–Menten constant
L-ASN	L-asparagine
L-ASNase	L-asparaginase
L-GTNase	L-glutaminase
MTT	3-[4,5-dimethylthiazol-2-yl]-2,5 diphenyl tetrazolium bromide
PBS	Physiological buffered saline
RPMI	Roswell Park Memorial Institute
SDS	Sodium dodecyl-sulphate
SDS-PAGE	Sodium dodecyl-sulphate polyacrylamide gel electrophoresis
*T*_1/2_	Half-life time
*T*_m_	Midpoint temperature
*V*_max_	Maximum velocity of reaction
